# Oxidative Stress in the Pathogenesis of Keratoconus and Fuchs Endothelial Corneal Dystrophy

**DOI:** 10.3390/ijms140919294

**Published:** 2013-09-23

**Authors:** Katarzyna A. Wojcik, Anna Kaminska, Janusz Blasiak, Jerzy Szaflik, Jacek P. Szaflik

**Affiliations:** 1 Department of Molecular Genetics, University of Lodz, Pomorska 141/143, 90-236 Lodz, Poland; E-Mails: kwojcik@biol.uni.lodz.pl (K.A.W.); jblasiak@biol.uni.lodz.pl (J.B.); 2 Department of Ophthalmology, Medical University of Warsaw, SPKSO Ophthalmic Hospital, Sierakowskiego 13, 03-709 Warsaw, Poland; E-Mails: anna.kaminska1@wum.edu.pl (A.K.); szaflik@szaflik.pl (J.S.)

**Keywords:** keratoconus, Fuchs endothelial corneal dystrophy, oxidative stress, antioxidants, free radicals

## Abstract

Due to its localization and function, the cornea is regularly exposed to sunlight and atmospheric oxygen, mainly dioxygen, which produce reactive oxygen species (ROS). Therefore, corneal cells are particularly susceptible to oxidative stress. The accumulation of ROS in the cornea may affect signal transduction, proliferation and may also promote cell death. The cornea has several enzymatic and non-enzymatic antioxidants involved in ROS scavenging, but in certain conditions they may not cope with oxidative stress, leading to diseases of the eye. Keratoconus (KC) and Fuchs endothelial corneal dystrophy (FECD) are multifactorial diseases of the cornea, in which pathogenesis is not fully understood. However, increased levels of oxidative stress markers detected in these disorders indicate that oxidative stress may play an important role in their development and progression. These markers are: (i) decreased levels of non-enzymatic antioxidants, and (ii) decreased expression of genes encoding antioxidative enzymes, including thioredoxin reductase, peroxiredoxins, superoxide dismutase, glutathione *S*-transferase, and aldehyde dehydrogenase. Moreover, the FECD endothelium displays higher levels of oxidative DNA damage, especially in mitochondrial DNA (mtDNA), whereas KC cornea shows abnormal levels of some components of oxidative phosphorylation encoded by mtDNA. In this review we present some considerations and results of experiments supporting the thesis on the important role of oxidative stress in KC and FECD pathology.

## 1. Introduction

The cornea is a transparent component of the eyeball, which along with eyelids, the tear film and the conjunctiva, protects the inner portion of the eye against external factors and pathogenic microbes. This tissue plays an important role in the refraction and transfer of light to the interior of ocular structures. Because of its function, the cornea is constantly exposed to light, including ultraviolet (UV) radiation. UV produces reactive oxygen species (ROS) which render the cornea particularly susceptible to oxidative stress as a consequence of an imbalance between ROS production and antioxidant capacity of the cell [1]. Therefore, oxidative stress reflects increased production and/or impaired removal of ROS. Reactive nitrogen species (RNS), especially those containing oxygen, may also contribute to stress. Moreover, it was demonstrated that UVA (wavelength range 315–400 nm), the main component of solar UV, induced bi-stranded oxidatively-induced clustered DNA lesions, which can be converted into DNA double strand breaks, when misrepaired [2]. This kind of DNA damage may play an important role in corneal damage and dysfunction. However, the cornea absorbs most of the UVB (280–315 nm) radiation reaching the eye, whereas UVA is primarily absorbed by the lens. Therefore, UVB seems to be the more important causative factor in molecular modifications that occur in corneal cells [3,4]. Despite this, UVA is particularly relevant for patients undergoing corneal cross-linking by the use of riboflavin and UVA; a treatment for keratoconus and other ecstatic disorders [5].

Although ROS and RNS are essential for physiological functions, including cell growth, proliferation, differentiation and apoptosis, their excess may have a detrimental influence on cellular components [6,7]. They can cause oxidative modifications of biomolecules, including lipids, proteins, carbohydrates and nucleic acids, leading to cellular and tissue damage [8]. Accumulation of ROS and RNS produced by external factors and normal cellular metabolism was associated with the development of several diseases, including: cancer, diabetes, autoimmune disorders, neurodegenerative diseases and aging [6,8,9]. Also, the cornea was shown to be affected by the accumulation of ROS and RNS, as well as due to malfunctions in corneal antioxidant defense mechanisms, leading to structural and functional changes in this tissue [10]. Oxidative damage has been associated with the pathogenesis of several ocular diseases, including age-related macular degeneration (AMD), cataract, uveitis, keratoconus (KC) and Fuchs endothelial corneal dystrophy (FECD) [11–15].

## 2. Oxidative Stress

**Figure 1:**
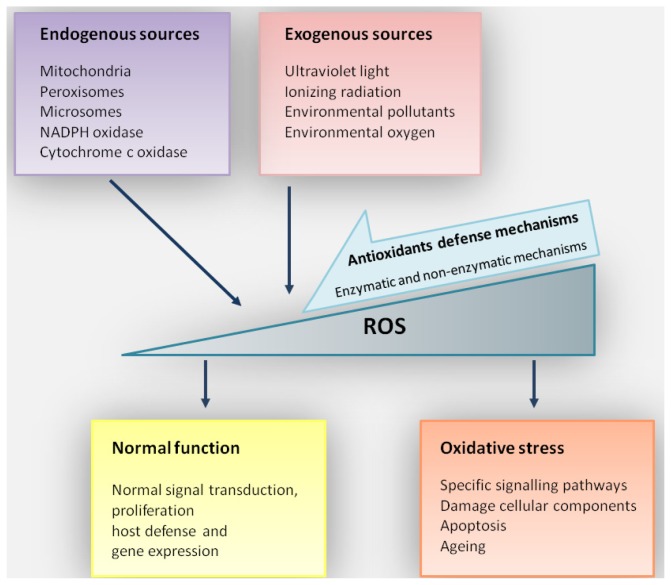
Cellular response to reactive oxygen species (ROS). ROS can be produced endo- or exogenously. The level of ROS, represented by grey triangle, is regulated by antioxidants defense mechanisms. Low levels of ROS production are required to maintain physiological functions, including: proliferation, host defense, signal transduction and gene expression. Under normal conditions, antioxidant defense mechanisms (including enzymatic antioxidants non-enzymatic molecules) decrease excess ROS and maintain cellular homeostasis. However, overproduction of ROS generates oxidative stress, leading to senescence, damage to cellular components, modulation of signal transduction pathways and apoptosis. Sometimes a reduced efficiency of antioxidant mechanisms may not be sufficient to decrease ROS level to physiological concentration.

Free radicals are formed as a result of normal cellular activity. These molecules play an important role in the regulation of diverse biological processes, including: signal transduction, proliferation, cellular metabolism, and gene expression. ROS and RNS participate in the immune response by the activation of macrophages and other phagocytes, as well as programmed cell death [6]. Furthermore, nitric oxide assists in leukocyte adhesion, angiogenesis, platelet aggregation and thrombosis [16]. Low concentrations of ROS and RNS are necessary for proper functioning of cells, tissues and whole organisms. However, ROS and RNS are also characterized by several detrimental actions, which may lead to pathological conditions [9,10]. Oxidative modification of biomolecules influences normal synthesis of proteins, DNA repair and several other processes, resulting in cellular dysfunction. Since mitochondria are the major site of oxygen metabolism, they are the most important source of endogenous production of ROS [8]. During oxidative phosphorylation, complex I and complex III in the mitochondrial inner membrane induce ROS as byproducts of their activity [17]. In addition, free radicals are generated in microsomes and peroxisomes, as well as via membrane-associated NAD(P)H oxidase, xanthine oxidase, and cytochrome *c* oxidase [6,18]. The presence of various transition metals, especially iron, contributes to ROS generation via the Fenton and Haber-Weiss reactions [19]. Nitric oxide synthase (NOS) isozymes catalyze the formation of nitric oxide that can be converted to other highly reactive RNS, including peroxynitrite and the nitroxyl anion [6,18]. However, ROS/RNS may also be generated by external agents, including UV, ionizing radiations and air pollution [7,18,20]. Cells and multi-cellular organisms evolved antioxidant defense mechanisms, including DNA repair, which neutralize ROS and repair DNA damage resulted from their action (Figure 1) [21]. Amongst several factors, these mechanisms have been reported to depend on tissue, age and the environment [7].

## 3. Oxidative Stress in the Pathogenesis of Keratoconus

**Figure 2:**
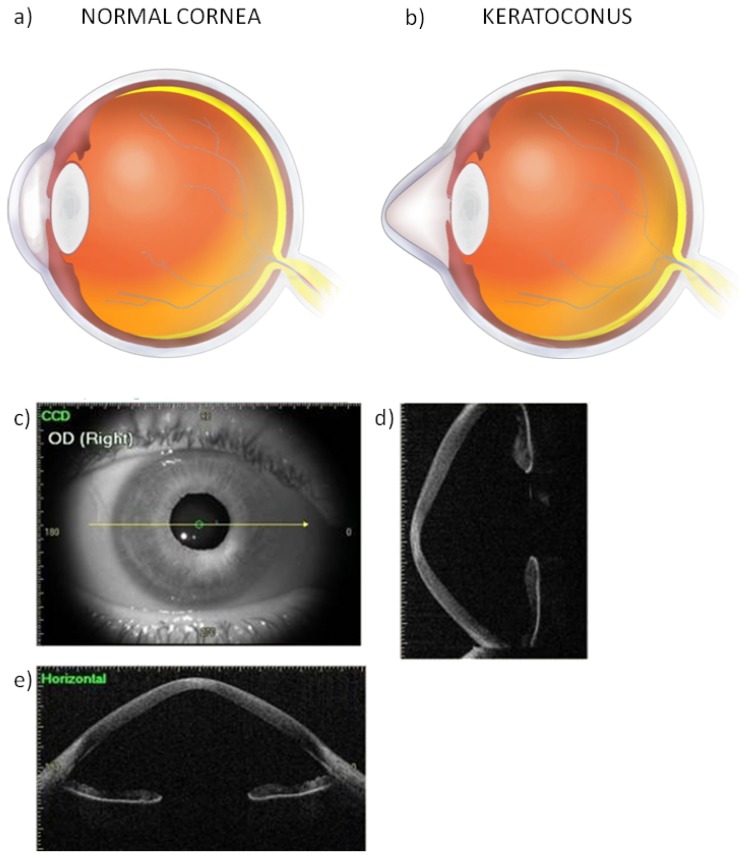
Diagrammatic comparison of normal cornea (**a**) and keratoconus cornea (**b**) along with images of keratoconus corneal thickness and elevation measurements obtained in swept-source optical coherence tomography (OCT) (**c**–**e**) of a patient from the Department of Ophthalmology, Medical University of Warsaw, Warsaw, Poland. The images present the vertical (**d**) and horizontal (**e**) images of OCT.

Keratoconus is a progressive corneal ectasia that occurs during the teen years and progresses until the fourth decade of life, when it typically stabilizes [22]. The prevalence of this disease is approximately 1 per 2000 in the general population [23]. KC is a bilateral disorder in which alternations in structure and function of the cornea depend on its progression [24]. It is characterized by central thinning of the corneal stroma, leading to deformation of the normal shape of the cornea (Figure 2) [25,26]. The disease is associated with breaks in Bowman’s layer and Fleischer’s ring–iron deposits in the basal layer of the epithelium [27]. Corneal protrusion causes irregular astigmatism and myopia, leading to decreased visual acuity [23].

The pathogenesis of KC is not completely known, but it is likely a multifactorial disease, with several genetic and environmental factors contributing to its development [24,28]. Eye rubbing, contact lens wear and atopy have also been reported among mechanical factors to be associated with the disease [29]. Although in the majority of cases, KC is reported as a sporadic disorder, autosomal recessive and autosomal dominant patterns of inheritance occur in some cases, suggesting a role of genetic factors in KC [30]. Numerous genetic analyses allowed for the identification of several candidate genes for KC [28]. Many of these studies suggest an association between KC and the visual system homeobox 1 (*VSX1*) gene, which encodes a member of the “paired-like” homeodomain transcription factors, important for ocular development [31,32]. However, several other studies did not confirm a correlation between *VSX1* and KC, indicating that other genetic factors may be involved [33]. Likewise, the superoxide dismutase 1 gene (*SOD1*), an important component of antioxidant defense, was proposed as a candidate associated with KC, but results of other studies failed to identify KC-related mutations in this gene [33]. Other reports indicate a significant role of collagen and products involved in its regulation in KC pathogenesis [28]. The *COL4A3* and *COL4A4* genes, encoding components of type IV collagen, a major corneal structural protein, were suggested as candidates in KC [34]. Mutations in the microRNA gene *MIR184,* expressed in the cornea and lens epithelium, were reported in KC with cataract [35]. Furthermore, genome-wide association studies indicated a link between KC and variations in and around the *HGF*, *RAB3GAP1* and *LOX* genes [28]. Despite identification of numerous genetic risk factors, KC genetics still needs to be elucidated. It was suggested that KC might involve changes in several genes (polygenic disease) [28].

A growing body of evidence indicates the role of oxidative stress in the development of KC [11,12]. Patients with KC were reported to have a higher level of free radicals and other reactive species in their corneas, than control samples [36,37]. Results of *in vitro* research on KC corneal fibroblast indicate an increased production of ROS and RNS, including superoxide, when compared with normal fibroblasts [36]. Furthermore, higher amounts of NOS and the accumulation of nitrotyrosine, a marker for the formation of peroxynitrite, were reported in KC corneas compared with normal samples [37]. An elevated amount of endothelial nitric oxide synthase (eNOS) at the site of Bowman’s layer breaks was observed in KC indicating an increased production of nitric oxide in these corneal regions [37]. NO and peroxynitrite may be implicated in multiple cytotoxic effects, including DNA damage and activation of apoptotic pathways [38]. Excessive RNS generation can contribute to modification of the stromal collagen molecules, increased apoptotic activity and KC corneal thinning. In addition, KC corneas were reported to have an elevated level of reactive aldehydes, such as malondialdehyde (MDA) and 4-hydroxy-2-nonenal (HNE), which may be produced by lipid peroxidation and may damage cellular biomolecules [37,39,40]. These aldehydes can also cause changes in the membranes of lysosomes, which in turn release proteolytic enzymes [41].

The level of ROS and RNS in normal corneas is regulated by antioxidant defense mechanisms, however results of some studies suggest disturbance in the level of transcripts and/or activities of different antioxidant enzymes in KC [30]. A decreased activity of extracellular superoxide dismutase (SOD) in KC corneas was recorded when compared to normal samples [42–44]. SOD is an important enzyme responsible for the dismutation of superoxide to oxygen and hydrogen peroxide. Alterations in the activity of SOD may lead to an increased amount of superoxide radicals [45]. Moreover, KC corneas exhibited a reduced level of aldehyde dehydrogenase Class 3 (ALDH3), that detoxifies reactive aldehydes produced by UV-induced lipid peroxidation [30,36,46].

Besides antioxidant enzymes, small molecular weight and non-enzymatic antioxidants are also involved in the protection of intracellular components against oxidative damage. This group includes reduced glutathione, which plays an important role in the regulation of cellular redox status and protection against free radicals and other oxidants [47]. A decreased glutathione content in KC cornea was reported when compared to normal samples [39].

Decreased antioxidant defenses in KC corneas, and increased levels of ROS and RNS may lead to the degradation of the extracellular matrix in the stroma and modification of cellular compounds, making them more susceptible to degradation, and thus result in thinner stroma in KC [11,39]. An influence of the nitric oxide pathway on the degradation of tissue inhibitors of matrix metalloproteinases (TIMP) was shown [43]. MMP are proteolytic enzymes that catalyze digestion of extracellular matrix components and other molecules on cell surface [48]. KC corneas had decreased mRNA and protein levels of TIMP-1, which in turn, led to an increase in matrix metalloproteinase 2 activity and corneal degradation [11]. Furthermore, TIMP-1 plays a role in the inhibition of apoptosis in a variety of cell types, therefore lower amount of this protein may be associated with fragmentation of the epithelium and thinning of KC cornea [25,49–51].

KC corneas exhibited an increased level of cathepsins V/L2, -B, and -G, which can stimulate hydrogen peroxide production, induce the degradation of collagen and proteoglycans, and lead to a chain reaction resulting in apoptosis [11,12,52]. Increased oxidative stress may damage lysosomal membranes, causing proteolytic enzymes release, triggering off stromal thinning [11,41].

Keratoconus corneas presented an increased level of mitochondrial DNA (mtDNA) damage, compared with age-matched normal corneas [53]. Human mtDNA is a covalently closed, double-stranded molecule, encoding 13 proteins of the oxidative phosphorylation chain, 22 tRNAs, and 2 rRNAs. mtDNA is located in close vicinity to the inner mitochondrial membrane, a major site of ROS production, therefore it is especially susceptible to oxidative damage [25]. DNA damage induced by oxidative stress may affect the protein-coding region of mtDNA and influence oxidative phosphorylation [54]. KC corneas exhibited a decreased activity of complex IV subunit 1 (CO I) in areas of corneal thinning [53]. Results of *in vitro* studies indicate an increased mitochondrial cytochrome oxidase subunit 2 (CO II) RNA levels in KC corneal stromal fibroblasts compared with normal cultures [12]. Aberrations in the expression of oxidative phosphorylation proteins may lead to improper ATP synthesis, increased ROS and RNS formation, and further oxidative damage [54,55]. Integrity of mtDNA plays an important role in viability of cells. An association between mitochondrial dysfunction and a variety of diseases was shown and it is hypothesized that mtDNA damage can also contribute to KC deformation [53,56].

Although excess ROS and RNS, disturbances in antioxidants defense mechanisms, as well as mitochondrial dysfunction have been detected in KC corneas, it is not clear which are the causes and which are consequences of the disease. Therefore, there is need for further studies focused on the involvement of oxidative stress in the pathogenesis of KC.

## 4. Oxidative Stress in the Pathogenesis of Fuchs Endothelial Corneal Dystrophy

**Figure 3:**
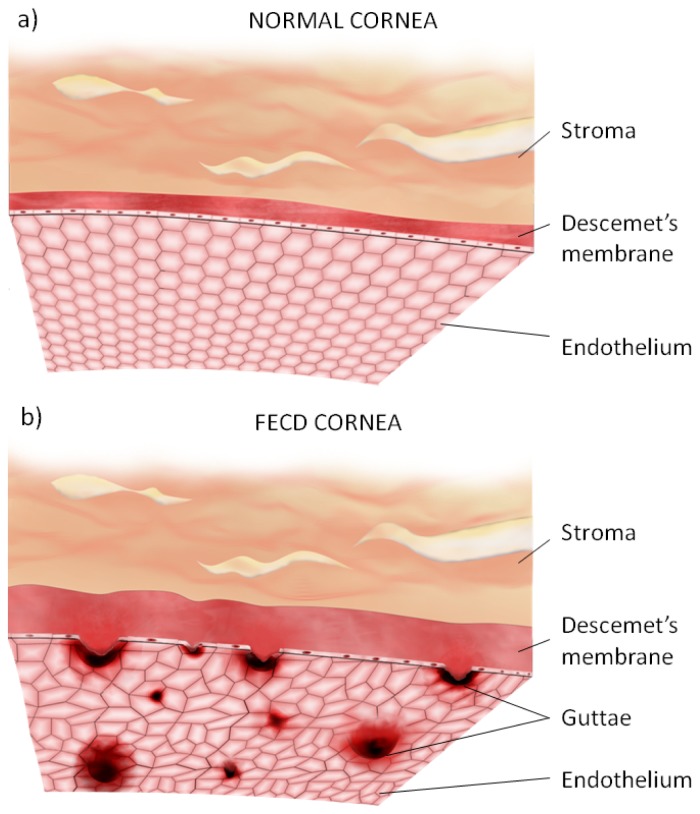

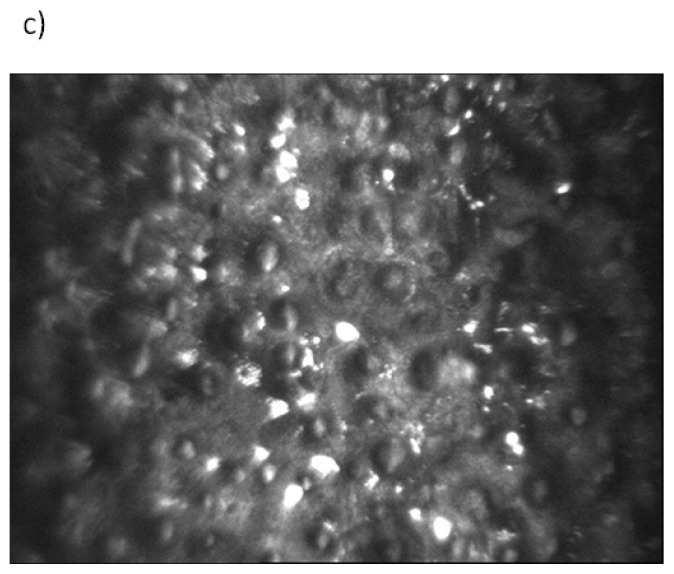
Endothelium and Descemet’s membrane from normal cornea (**a**) and cornea in Fuchs endothelial corneal dystrophy (FECD) (**b**) showing structural changes, including guttae formation, modification of hexagonal endothelial cell mosaic and Descemet’s membrane thickening in FECD. Representative confocal microscopy image of endothelial guttae in advanced stage of FECD obtained from a patient of the Department of Ophthalmology, Medical University of Warsaw, Warsaw, Poland, is shown in part (**c**).

FECD is a disorder caused by degeneration of the corneal endothelium. The disease typically affects both eyes in the fourth or fifth decade of life [57]. It affects approximately 4% of the population over 40 years of age. However, the incidence of FECD is higher in the European population compared to other parts of the world [58]. FECD is characterized by the formation of excrescences in the Descemet’s membrane called cornea guttae (Figure 3) [59,60]. In the early stage of the disease, guttae are located in the central cornea. As the disease progresses, excrescences grow and spread towards the periphery cornea with concomitant loss of hexagonal shape and density of endothelial cells [61,62]. Corneal endothelium plays an important role in the regulation of transport of the corneal stromal fluid [63]. Endothelial cells regulate the uptake of solutes and nutrients by the cornea *via* diffusion and secondary active transport mechanisms [61,64]. Furthermore, ion transport in the endothelium is responsible for fluid secretion from the stroma to the aqueous [64]. In the physiological state, leak of fluid into the cornea is continuously counterbalanced by the fluid transport that keeps the cornea in a relatively dehydrated state in order to sustain its transparency. Loss of endothelial cells density in FECD triggers loss of endothelial ion transport activity, causing their inability to maintain a proper fluid balance resulting in stromal and epithelial edema and subsequent loss of the clarity of the cornea [61,64]. Corneal dysfunction in FECD is manifested by progressive decrease in visual acuity and may lead to blindness [59].

Although autosomal dominant pattern of inheritance in FECD was reported, the precise mode of perpetuation for most cases of the disease remains unknown [65]. FECD is considered as a multifactorial disease, in which genetic, as well as environmental factors, play a role in its development [61]. Familial form of FECD is associated with the mutations in the *COL8A2* gene, encoding α2 subtype of collagen VIII, which is a major component of Descemet’s membrane [66]. Other reports suggest a role of heterozygous mutation in the *SLC4A11* gene in the development of sporadic cases of the disease [57]. Moreover, *TCF8,* encoding a zinc finger transcription factor, was considered as a candidate gene for the disease [67]. Family-based studies allowed identification of 13pTel-13q12.13 and 18q21.2-q21.32 as FECD susceptibility *loci* [61]. Moreover, genome wide linkage analysis of patient with familial FECD identified potential linkage regions on chromosomes 1, 7, 15, 17, and X [62]. Although genetic studies showed several genes, as well as chromosomal loci associated with the disease, the precise role of these regions in FECD pathogenesis is still unknown and requires further investigation.

Results of several studies indicate an association between FECD and oxidative stress. FECD corneas exhibited an accumulation of ROS and RNS products, which were not detected in normal human corneas [37]. Moreover, FECD corneas showed an increased level of nitric oxide synthase compared with normal tissues, which indicates an increase in nitric oxide production associated with FECD [37].

An aberrant expression of many proteins, including antioxidant enzymes, was reported in FECD endothelial cells. FECD cells showed downregulation of glutathione *S*-transferase, ALDH3A1 and ferritin [68]. Proteomic analyses indicated a decreased expression of peroxiredoxins in FECD corneal endothelial cells and Descemet’s membrane compared with the control [69]. Peroxiredoxins are involved in the removal of hydrogen peroxide from the cells, and the inhibition of ROS-induced apoptosis. In addition, FECD corneas displayed a decreased expression of the heat shock 70-kDa protein that plays an important role in the protection against apoptosis [68,70]. Furthermore, FECD endothelium displayed a decreased level of SOD2 in the mitochondrial matrix, metallothionein 3 (MT3), and thioredoxin reductase 1 (TXNRD1) [13]. TXNRD1 catalyze the reduction of thioredoxin that keeps metallothionein 3 and peroxiredoxins in the reduced state [13,71]. This protein also catalyzes the regeneration of various low molecular weight antioxidants, including vitamin E, vitamin C, lipoic acid and superoxide [72]. Thioredoxin reductase is involved in the regulation of some transcription factors, which control the expression of many genes important for the proper functioning of cells [71,73]. Downregulation of antioxidant enzymes in FECD may lead to perturbation of tissue homeostasis and activation of apoptotic pathways [68].

Promoter regions of several antioxidant and xenobiotic-metabolizing enzyme genes contain the antioxidant responsive element (ARE), involved in the regulation of expression of oxidative stress-related genes, including glutathione *S*-transferases, heme oxygenase-1 (HO-1), peroxiredoxin, TXNRD1, thioredoxin, and ferritin [74,75]. The transcription factor Nrf2 shows a high affinity to the ARE sequence and may be involved in transcriptional activation of genes encoding proteins important for the protection against oxidative stress [74,75]. A decrease in the Nrf2 protein level in FECD endothelium was observed. In addition, a decreased expression of the *HO-1* gene, which is one of the major Nrf2-regulated antioxidant genes, was shown. Aberrant Nrf2 response may influence the expression of multiple antioxidants in FECD corneal endothelium, causing accumulation of free radicals and other reactive species [13,62]. Increased oxidative stress in FECD cornea may contribute to endothelial oxidative DNA damage, morphological modification and apoptosis. We showed that FECD patients displayed greater levels of oxidative DNA lesions compared with age- and sex-matched controls [76]. It was also shown that the level of 8-hydroxy-2′-deoxyguanosine, a marker of oxidative DNA damage, was increased in FECD endothelium [13]. The majority of DNA damage was located in mtDNA. These findings suggest that mtDNA is the primary target of oxidative injury in FECD [13]. A decreased numbers of mitochondria in the FECD endothelium as well as reduced activity of cytochrome oxidase, the major respiratory chain enzyme, in the central areas of FECD corneal buttons, were also shown in the same study [13]. Several studies suggest association between mtDNA damage and aging [77] where senescent cells accumulate oxidative damage to mtDNA. Therefore, the progressive oxidative damage in mtDNA can lead to modification of the coding region of genes encoding components of respiratory chain and alter their expression, which may result in disturbances in respiratory chain function and further production of ROS and RNS [78]. Furthermore, the mitochondrial dysfunction can cause loss of integrity of inner mitochondrial membrane potential and apoptotic cell death [55]. An increased level of oxidative DNA damage next to corneal guttae was reported, indicating an association between macromolecular damage triggered by oxidative stress and apoptosis of the endothelial cells in FECD [13].

## 5. Conclusions

**Figure 4:**
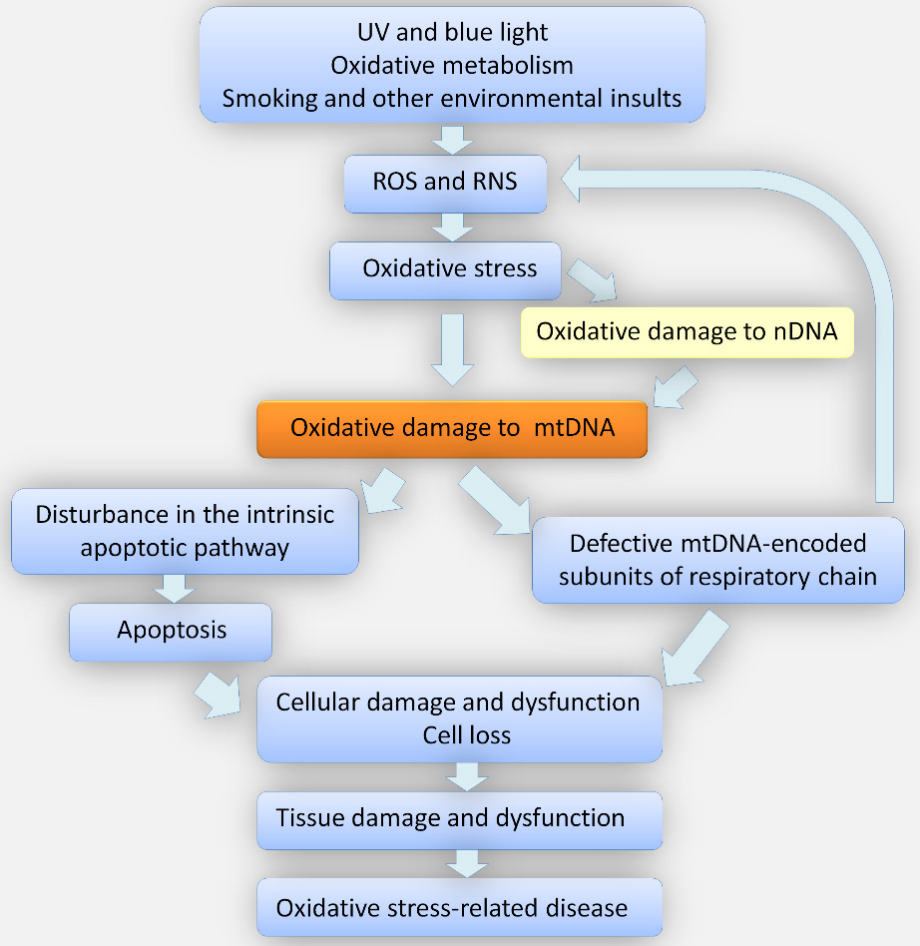
Oxidative stress may result from endogenous and exogenous processes and is associated with overproduction of reactive oxygen/nitrogen species (ROS/RNS), which may damage both mitochondrial (mt) and nuclear (n) DNA. This may cause changes in several pathways associated with cell survival and functionality, which may contribute to a disease phenotype. See text for more details.

Environmental factors, such as UV radiation, may induce ROS/RNS, which in turn induce oxidative stress, resulting in further formation of reactive species, which may damage DNA (Figure 4). Mitochondrial DNA is especially prone to such damage due to its close proximity to the respiratory chain. However, the reaction of mtDNA to damage-inducing factors is mainly determined by proteins encoded by nuclear DNA. In turn, mtDNA damage may result in defective functioning of oxidative chain protein(s), producing an excess of ROS/RNS and increasing the extent of oxidative stress. On the other hand, damaged mtDNA may disturb signaling of the intrinsic apoptotic pathway. Altogether, damage to mtDNA and its nuclear counterpart caused by oxidative stress may lead to cellular damage and cell loss, resulting in tissue damage and dysfunction, and finally contributing to disease phenotype, including KC and FECD.

Despite many extensive studies, the pathogenesis of KC and FECD are poorly understood, which is probably underlined by the complexity of these diseases. The role of oxidative stress in both diseases needs further investigation. It is still not known whether oxidative stress in KC and FECD corneas is the direct cause of the diseases, or whether it is a consequence of disturbed functioning of the corneal cells.
